# S100A9-Imaging Enables Estimation of Early Therapy-Mediated Changes in the Inflammatory Tumor Microenvironment

**DOI:** 10.3390/biomedicines9010029

**Published:** 2021-01-03

**Authors:** Anne Helfen, Annika Schnepel, Jan Rieß, Miriam Stölting, Mirjam Gerwing, Max Masthoff, Thomas Vogl, Johannes Roth, Carsten Höltke, Moritz Wildgruber, Michel Eisenblätter

**Affiliations:** 1 University Clinic of Radiology, Medical Faculty, University of Muenster and University Hospital Muenster, D-48149 Muenster, Germany; a_schn52@uni-muenster.de (A.S.); j_ries08@uni-muenster.de (J.R.); Miriam.Stoelting@ukmuenster.de (M.S.); Mirjam.Gerwing@ukmuenster.de (M.G.); Max.Masthoff@ukmuenster.de (M.M.); carsten.hoeltke@uni-muenster.de (C.H.); Moritz.Wildgruber@med.uni-muenchen.de (M.W.); michel.eisenblaetter@uniklinik-freiburg.de (M.E.); 2 Institute of Immunology, University of Muenster, 48149 Muenster, Germany; vogl@uni-muenster.de (T.V.); rothj@uni-muenster.de (J.R.); 3 Department for Radiology, University Hospital, LMU Munich, D-81377 Munich, Germany; 4 Department of Diagnostic and Interventional Radiology, Medical Center—University of Freiburg, D-79106 Freiburg, Germany

**Keywords:** calprotectin, MRP8/MRP14, tumor-associated monocytes, tumor microenvironment, molecular imaging

## Abstract

(1) Background: The prognosis of cancer is dependent on immune cells in the tumor microenvironment (TME). The protein S100A9 is an essential regulator of the TME, associated with poor prognosis. In this study, we evaluated early therapy effects on the TME in syngeneic murine breast cancer via S100A9-specific in vivo imaging. (2) Methods: Murine 4T1 cells were implanted orthotopically in female BALB/c mice (*n* = 59). Tumor size-adapted fluorescence imaging was performed before and 5 days after chemo- (Doxorubicin, *n* = 20), anti-angiogenic therapy (Bevacizumab, *n* = 20), or placebo (NaCl, *n* = 19). Imaging results were validated ex vivo (immunohistochemistry, flow cytometry). (3) Results: While tumor growth revealed no differences (*p* = 0.48), fluorescence intensities (FI) for S100A9 in Bevacizumab-treated tumors were significantly lower as compared to Doxorubicin (2.60 vs. 15.65 AU, *p* < 0.0001). FI for Doxorubicin were significantly higher compared to placebo (8.95 AU, *p* = 0.01). Flow cytometry revealed shifts in monocytic and T-cell cell infiltrates under therapy, correlating with imaging. (4) Conclusions: S100A9-specific imaging enables early detection of therapy effects visualizing immune cell activity in the TME, even before clinically detectable changes in tumor size. Therefore, it may serve as a non-invasive imaging biomarker for early therapy effects.

## 1. Introduction

Tumor growth, invasion, and metastasis are strongly influenced by tumor-infiltrating immune cells, which form a characteristic inflammatory tumor microenvironment (TME). In breast cancer, high local numbers of regulatory T cells, immature monocytic cells, and natural killer cells are associated with poor prognosis and reduced survival [1].

Within the TME, the protein complex S100A8/S100A9 is expressed and released by cells of granulocytic and monocytic origin, such as tumor-associated macrophages (TAM) supporting vascular invasion and neoangiogenesis by degrading the extracellular matrix and by inducing myeloid-derived suppressor cells (MDSC) [2,3]. In previous studies, an expression of the protein complex by tumor cells themselves, at least for 4T1 breast cancer, could be excluded [4]. The non-covalently associated heterocomplex S100A8/S100A9 is physiologically involved in the regulation of different cellular processes, such as differentiation and migration. After secretion via an unknown mechanism as a heterodimer, S100A8/S100A9 induces proinflammatory effects binding Toll-like receptor 4 or the receptor for advanced glycation end products regulating migration, activation, and maturation of myeloid cells [5,6]. Early changes in the cellular composition under inflammatory conditions, including the TME, are reflected by an increase in local S100A8/S100A9 expression [6]. The protein complex S100A8/S100A9 is an important promoter of tumor invasiveness and has been associated with poor prognosis [7].

In previous studies, S100A9-specific (=S100A8/S100A9) in vivo imaging for the detection of local monocytic activity in the TME as a surrogate for tumor aggressiveness and development has been established [4], while beforehand evaluation of S100A8/S100A9 and MDSC in humans and mice was focused on ex vivo analyzes from peripheral blood and lymphatic organs.

Methods for the early assessment of treatment response and prognosis in cancer patients undergoing highly specific, personalized therapy are still largely missing, as the effects on the tumor, resulting in measurable changes in morphology and size, may be delayed or absent [8]. In this context, a non-invasive imaging method to visualize immunological changes, not the tumor within the TME, as effects of tumor treatment could circumvent these hurdles, offering much-needed information as to treatment response or failure [9]. It has already been proposed for breast cancer that response of cancer cells to specific drugs is not exclusively determined by the tumor’s intrinsic characteristics but is also controlled by the TME [10]. Components of the TME also have pivotal roles in determining treatment outcome as immunological changes are induced in the TME and may promote tumor recurrence after radiation therapy [11,12]. In this context, tools for dynamic mapping of the cellular composition of the TME could be essential for the estimation of the treatment response, prognosis, and risk of tumor recurrence.

The aim of this study, therefore, was to investigate the potential of S100A8/S100A9-specific imaging for the estimation of very early treatment effects on the inflammatory TME, even before they are clinically detectable. 

In a syngeneic murine breast carcinoma model, changes in monocytic activity and immune cell composition within the TME have been investigated in two different treatment cohorts: one group receiving conventional, cytotoxic chemotherapy, the second group receiving an anti-vascular endothelial growth factor (VEGF) antibody, inhibiting neoangiogenesis and thus counteracting monocyte activity in the TME. In a third cohort, sodium chloride as a placebo served as the control for unspecific effects.

## 2. Experimental Section

### 2.1. Tumor Model

All animal experiments in this study were approved by the responsible authorities (Protocol No. 84-02.04.2017.A011). 4T1 murine breast cancer was chosen as a syngeneic tumor model in BALB/c mice with a high malignant potential (rapid local growth, lymphatic and hematogenous metastasis). Female BALB/c (Charles River Laboratories, Germany) mice (*n* = 59, age: 8–12 weeks) were implanted orthotopically with 0.5 × 10^6^ tumor cells in 50 μL of phosphate-buffered saline via injection into the lower-left mammary fat pad. Extra-fine 29G syringes and no additives to the cell solution were used to reduce trauma and consecutive inflammation to an absolute minimum.

**Figure 1:**
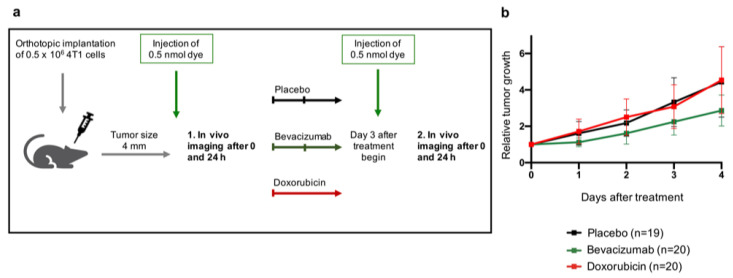
Therapy scheme and relative tumor growth under therapy. (**a**) Experimental setup including the three different treatment regimen (placebo: *n* = 19, Bevacizumab: *n* = 20, Doxorubicin: *n* = 20). (**b**) Comparison of relative tumor growth (=difference of absolute tumor size compared to therapy begin) between the three different treatment groups revealed no significant differences during the first 4 days after therapy began until the second in vivo imaging.

Maximum diameters of tumors were measured daily using a digital caliper. All imaging experiments were performed size-dependent, once tumors reached a diameter of 4 mm (tumor volume approximately 268 mm^3^ (4/3 × π × 4 mm)), to control for tumor size effects. Relative tumor growth was calculated after the baseline imaging with the start of treatment (day 0) until the final second imaging on day 4; the respective ratio was formed to size at day 0 (see Figure 1).

After the second in vivo imaging, mice were sacrificed, and tissue was harvested for flow cytometry and histological analysis as described below.

### 2.2. Imaging Probe and Imaging Procedure

For imaging of S100A9, a polyclonal antibody, binding the S100A9 subunit of the complex was labeled with the cyanine dye Cy5.5 (anti-S100A9-Cy5.5; λex/em 678/696 nm, GE Healthcare Bio-Sciences Corp.), as described in detail earlier [13].

Briefly, the antibody was purified using size exclusion chromatography, and an amount of 1.5 mg was referred to labeling with Cy5.5 according to the dye manufacturer’s protocol. The labeled antibody was purified from a free dye by a PD10 column. Photometric analysis revealed an average labeling ratio of three dye molecules per antibody for the batch that was consecutively used in this study. The tracer doses per animal were normalized for the fluorescent dye: per imaging session, each mouse received tracer equivalent to 0.5 nmol Cy5.5.

To evaluate the probe’s specificity and sensitivity, a polyclonal rabbit IgG was labeled with Cy5.5 accordingly (*n* = 5).

Mice received the fluorescent dyes within a single intravenous injection once the individual tumors reached the aforementioned size threshold. Immediately before (0 h) and 24 h after tracer administration and shaving the abdominal region, mice underwent fluorescence reflectance imaging (FRI; Bruker BioSpin). Excitation was chosen at 630 nm, and the resulting emission was recorded at 700 nm using a filter-equipped, high-sensitive charge-coupled device camera. Signal acquisition time was 5 s. For anatomical colocalization, white light images were obtained. During examination, mice were held under inhalation anesthesia (isoflurane 2.0% in 2 L/min air during examination; 2.5% for 2 min induction, immediately before imaging).

On the in vivo imaging data, the regions of interest (ROIs) for further image analysis were chosen to cover the whole tumor area as depicted on white light images. FRI data are presented as mean photon counts within the defined ROI in arbitrary units (AU). The comparison of the fluorescence intensities is given as the dynamic development of contrast-to-noise-ratios with healthy tissue of the contralateral side as reference (CNR = (mean photon counts normalized for the ROI in the tumor-mean photon counts normalized for the ROI in the healthy tissue of the contralateral side)/standard deviation of the background) between the measurements immediately before (0 h) and 24 h after probe injection as “CNR 24 h—0 h”.

### 2.3. Therapy Scheme and Tumor Growth

After the first tumor size-dependent in vivo imaging, mice were randomly assigned to the three treatment groups following the experimental design shown in Figure 1a and were treated over a period of 4 days. The selected dosages were based on established human clinical doses for (adjuvant) treatment of breast cancer and were used in coordination with the local animal welfare authorities as well as previously described in preclinical studies. One group (*n* = 20, imaging *n* = 16) received a single Doxorubicin bolus (5 µg/g body weight, adapted from [14,15]) intravenously as a conventional chemotherapeutic agent (anthracycline). A second group (*n* = 20, imaging *n* = 15) was treated with the anti-VEGF-antibody Bevacizumab twice (5 µg/g body weight, injection on day 0 and 2, adapted from [16]). Sodium chloride (NaCl 0.9%) solution (5 µg/g body weight, injection on day 0 and 2, adapted to the dosages of Doxorubicin and Bevacizumab) served as a placebo in a third group (*n* = 19, imaging *n* = 12) and was injected intravenously twice (see also Supplementary Table S1 for therapy groups and dosages).

Concerning the relative tumor growth, the tumor size increased continuously in all treatment groups. Albeit the growth rate was diminished under Bevacizumab treatment, differences between the applied study treatment groups were not accessible visually and not statistically significant (*p* = 0.48), as shown in Figure 1b. For this reason, the focus of the observations in this study was placed on the evaluation of the in vivo imaging as an early differentiating parameter for therapy response.

### 2.4. Ex vivo Correlation and Immunohistochemistry

Immediately after the second in vivo imaging, mice, still under anesthesia, were sacrificed by cervical dislocation. Tumor tissue was harvested for histology or flow cytometry. For immunohistochemistry, tumors were paraffin-embedded and cut into 5 μm slices. Staining for S100A9 was performed, using the same antibody as for in vivo imaging, following established protocols [4].

For flow cytometry, single-cell suspensions were produced from tumor tissue and stained for CD 11b, Ly6C, CD 3, CD 4, and CD 8 with corresponding isotype controls as listed in Table 1.

Cell debris was excluded from flow cytometry analysis, filtering for size, and granularity. All measurements were performed using a FACSCalibur system (Becton Dickinson, Franklin Lakes, NJ, USA) and analyzed using FlowJo software (FlowJo LLC, Ashland, Oregon). The data were presented as event frequencies, which were reduced by the individual isotype control to exclude non-specific staining (gating strategy in Supplementary Figure S2).

### 2.5. Statistical Analysis

Data were analyzed using a Mann–Whitney–Wilcoxon test or a one-way ANOVA with Bonferroni post-correction. Analyzes were performed using the GraphPad Prism Software (version 8.4.3; GraphPad Software Inc., San Diego, California, USA). A *p*-value of 0.05 or lower was considered significant.

## 3. Results

### 3.1. In Vivo Imaging

**Figure 2:**
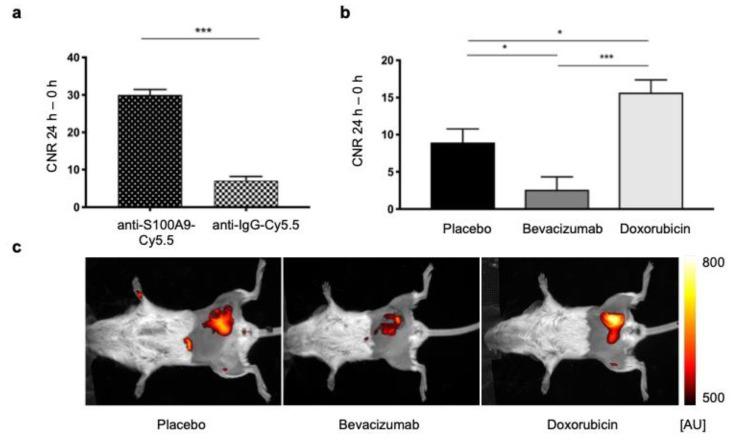
S100A9-specific in vivo Fluorescence Reflectance Imaging. (**a**) Validation of the S100A9-probe´s specificity: (**b**) Comparison of CNR after treatment (second in vivo imaging): Compared to placebo, fluorescence intensities under Doxorubicin were significantly higher (*p* = 0.01) and under Bevacizumab significantly lower. Furthermore, significant differences between the Bevacizumab and the Doxorubicin group were detected. (**c**) Exemplary fluorescence reflectance imaging (FRI) images (overlay of white light and fluorescence images) 24 h after probe injection. The visual comparison of the fluorescence intensities correlates with the measured values shown in (**b**). Asterisks indicate significance: * *p* < 0.05, *** *p* < 0.001.

To prove the imaging probe’s suitability for visualization of the TME, in a first validation step, the target specificity of the synthesized anti-S100A9-Cy5.5 probe was confirmed: The CNR of anti-S100A9-Cy5.5 (*n* = 46, before treatment) was significantly higher as compared to the unspecific anti-IgG-Cy5.5 (*n* = 5, 30.03 vs. 7.06, *p* < 0.0001, Figure 2a).

While there were no significant macroscopic differences in terms of tumor growth, fluorescence reflectance imaging revealed significant alterations of CNR already in the early course of 4 days after the start of therapy (Figure 2b,c). CNR in tumors under treatment with Doxorubicin was significantly higher than in the placebo group (15.65 vs. 8.95, *p* = 0.01). Compared with placebo, tumors of the Bevacizumab group had a significantly lower CNR (2.60 vs. 8.95, *p* = 0.02). Furthermore, early FRI imaging enabled distinguishing both treatment groups: CNR in the Doxorubicin group was significantly higher as compared to the Bevacizumab group (15.65 vs. 2.60, *p* < 0.001).

### 3.2. Immunohistochemistry

**Figure 3:**
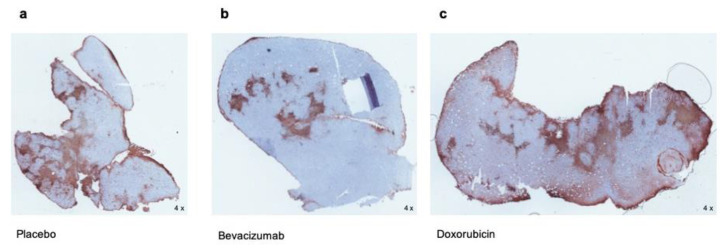
Immunohistochemistry for S100A9. Microscopic overview images (4-fold magnification) of paraffin-embedded whole tumor slices. Staining with an anti-S100A9-antibody (positive cells in brown). Visually inspected, the number of S100A9-positive cells correlates with the respective fluorescence intensities of the different treatment groups under (**a**) placebo, (**b**) Bevacizumab, and (**c**) Doxorubicin.

Immunohistochemistry for S100A9 confirmed the data obtained by means of FRI in vivo. Within the whole tumor slices, S100A9-positive cells (stained brown) correlated with the CNR determined for the therapy groups via FRI (Figure 3). S100A9 positive cells were most abundant under treatment with Doxorubicin (Figure 3c) and fewest under Bevacizumab (Figure 3b).

### 3.3. Fluorescence-Activated Flow Cytometry

**Figure 4:**
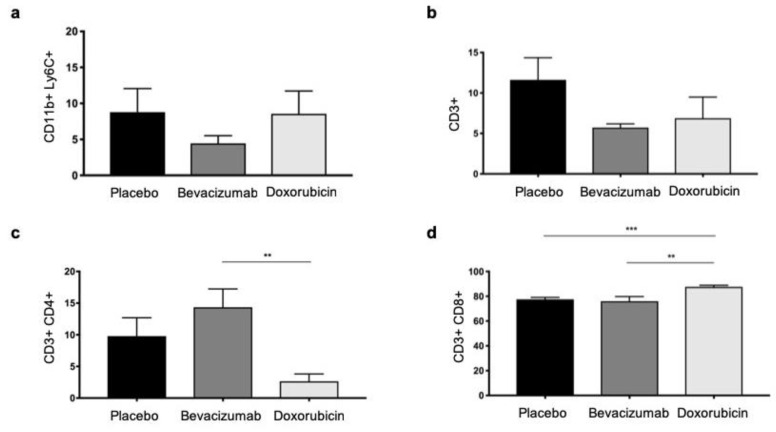
Flow cytometry analyses of the tumor microenvironment (TME). (**a**) The amount of immature myeloid cells (CD11b+/Ly6C+) was similar to the distribution of fluorescence intensities of anti-S100A9-Cy5.5. However, the results were not significant. (**b**) Concerning T cells (CD3+, b), Bevacizumab and Doxorubicin treated tumors had lower cell amounts as compared to placebo. (**c**) T helper cells (CD3+/CD4+) were significantly higher under Bevacizumab as compared to Doxorubicin (*p* = 0.005). (**d**) Amounts of cytotoxic T cells (CD3+/CD8+) were significantly elevated under Doxorubicin treatment as compared to Bevacizumab (*p* = 0.009) as well as placebo (*p* < 0.001). Asterisks indicate significance: ** *p* < 0.01, *** *p* < 0.001.

The flow cytometry analyzes for CD11b (pan-myeloid innate immune marker predominantly expressed by monocytes, macrophages, granulocytes, and dendritic cells) and Ly6C (marker for monocytes), as well as the S100A9-positive immunohistochemistry, correlated with the imaging results, supporting the hypothesis, that myeloid cells, in particular, are involved in the production of S100A9 within the TME. As with S100A9-specific imaging, the number of CD11b and Ly6C positive cells in tumors was lower under Bevacizumab (*n* = 9) compared to the placebo group (*n* = 10) and increased under Doxorubicin (*n* = 6), however differences were not significant (*p* = 0.6, Figure 4a).

In addition to the therapeutic influence on monocytic cells in the TME, a possible influence on the T cell composition was also investigated using flow cytometry. The total number of CD3 positive T cells was lower under both Bevacizumab and Doxorubicin compared to placebo, although not significant (Figure 4b).

However, there were differences with regard to the subsets of the T cells. The number of T helper cells, promoting tumor growth under the influence of the TME (CD4+), was significantly increased under Bevacizumab as compared to Doxorubicin (*p* = 0.005). Under Doxorubicin, on the other hand, the cell count was also lower than with the placebo, however not significantly (Figure 4c).

In contrast, the cytotoxic T cells made up by far the largest proportion of the total number of CD3 positive cells with over 70%. Under Doxorubicin, their number within the TME was significantly increased as compared to both placebo (*p* < 0.001) and Bevacizumab (*p* = 0.005, Figure 4d).

## 4. Discussion

Current cancer therapy monitoring largely relies on imaging of tumor size. Increasingly individualized tumor medicine with therapy not necessarily affecting the tumor morphology demands a paradigm shift to overcome the lack of early indicators for therapy response [8]. The protein complex S100A8/S100A9 has been associated with poor tumor prognosis [7]. The protein complex primarily represents the local activity of TAM and MDSC in the TME. Its higher expression, therefore, reflects various cellular changes within the TME and correlates with tumor aggressiveness.

In the present study, differences in S100A8/S100A9 expression in the TME, reflective of shifts in the immunological composition of the TME could be detected in the very early course of therapy via in vivo imaging, when macroscopic differences in the form of tumor growth, which, therefore, would also be depictable via clinically established imaging methods, were not yet evident. The observation of a lack of measurable early changes in tumor growth also coincides with previous preclinical therapeutic studies of Doxorubicin [15,17,18] and Bevacizumab [19,20] and respective control groups. The non-invasive in vivo imaging of S100A9, as described here, reflects the local activity of the TME during tumor development as well as changes under treatment. Since S100A8/A9 is not released by the tumor cells themselves, at least not in the case of murine 4T1 breast cancer [4], there is a number of implications.

Since imaging was performed at a very early time point after therapy onset, no relevant tumor cell apoptosis had yet occurred, as represented by the nonsignificant differences in tumor growth. Therefore it is likely that the presented differences in in vivo imaging take place at a subcellular level within the TME.

In the current study, local S100A8/S100A9 levels were significantly increased in the TME both in vivo and ex vivo under Doxorubicin treatment as compared to the placebo group. Doxorubicin mediates immunogenic cell death by translocating calreticulin from the cytoplasm to the cell surface, stimulating phagocytosis of tumor cells by dendritic cells. Further, activation of TLR-4 on dendritic cells simulates T cells [21], which are, under the influence of Doxorubicin, also recruited via IFN-γ [22]. At the same time, IFN-γ activates myeloid-derived suppressor cells (MDSC), which might lead to the observed elevated S100A9 expression [23] since S100A9 is not released during therapy-induced lysis of the tumor cells.

Furthermore, intrinsic chemoresistance through upregulation of p-glycoprotein and IL-6 has been described for 4T1 tumors [24]. The chemoresistance can also be mediated by overactivation of CXCL1 and 2, which exert prometastatic effects through increased expression of S100A9 [25]. The selection and survival of chemo resistant tumor cells are enhanced in this way. At the same time, this observation is another possible explanation for an increase in S100A9 under Doxorubicin.

Under treatment with Bevacizumab, the S100A9-specific fluorescence signal was significantly lower within the TME both in vivo and ex vivo, as compared to placebo. Within the TME, VEGF increases the accumulation of MDSC. The anti-VEGF antibody Bevacizumab reduces the amount of MDSC and improves the maturation of dendritic cells, thus increasing their amount within the TME and supporting anti-tumoral effects [26]. These findings may explain the lower amount of S100A9, mainly expressed and released by myeloid cells, such as MDSC. Under combined clinical treatment with Bevacizumab and a tyrosine kinase inhibitor, lower amounts of S100A9-positive cells, and accordingly in non-small-cell lung cancer, a significant reduction in granulocytic MSDC, were also found in human blood samples, underlining the aforementioned preclinical findings [27,28].

Under treatment with Doxorubicin, the amount of cytotoxic CD8+ cells increased in our study. The antitumor effect of Doxorubicin is decisively regulated by CD8+ cells and IFN-γ. Local application of the therapeutic agent promotes the maturation and accumulation of CD8+ cells [22,29]. Similarly, activation of cytotoxic T cells following an IL-2 increase after intraperitoneal application of Doxorubicin in rats has been described previously [30]. Camilio et al. examined the 4T1 breast cancer model under monotherapy with Doxorubicin and described an increase in CD8+ cells 6 days after therapy onset, matching our observations. At this time point, CD4+ cells were decreased, followed by an increase during the subsequent longer-lasting therapy period as compared to our experimental setting [31]. It can, therefore, be assumed that at the time selected in our study, it was too early to observe the described increase in CD4+ cells.

With regard to the changes under Bevacizumab therapy, the hypothesis exists that under physiological conditions VEGF inhibits the maturation of dendritic cells as well as T cells [32,33]. Therefore, anti-VEGF therapy could result in an increase in the T cell population.

Regarding the present study, a few limitations were observed. In principle, an observation over a longer period would be very desirable to be able to measure longitudinal changes in the TME. However, due to animal welfare regulations, follow-up of mice after tumor treatment was limited to the chosen time points. The results presented here provide indications for changes in the T cell population under the different forms of therapy. Ultimately, however, more differentiated flow cytometry analyzes would be necessary to differentiate further expanded T helper cell populations and regulatory T cells. Additionally, genomic and proteomic analyzes of the different T cell populations would be desirable. It should also be noted that in the clinical context, Bevacizumab is not used as a monotherapy in the clinical setting but always in combination with other anticancer agents. Nevertheless, in this study, we chose the monotherapeutic approach to be able to evaluate the specific effects on the TME.

## 5. Conclusions

The S100A9-specific in vivo imaging presented here allows the assessment of changes in the natural TME non-invasively and longitudinally over time, reflecting immediate and divergent therapy effects, even before the size or morphology of the tumor are responding. With S100A8/S100A9 as an important mediator in the TME, this study can pave the way for an imaging biomarker development for modern tumor therapy and ultimately underline its translational potential.

## Figures and Tables

**Table 1:** Flow cytometry staining strategy for examined antibodies (first column) with corresponding isotype controls (fourth column) and addressed cell types (third column).

Addressed Cell Types	Antibody	Clone/Origin of Antibody	Isotype Control	Clone/Origin of Isotype Control
dendritic cells, monocytes, granulocytes	Cd11b-PB	M1/70/BioLegend, San Diego, California, USA	rat-IgG-2b,κ-PB	RTK4530/BioLegend, San Diego, California, USA
premature monocytes	Ly6C-PE	HK1.4/BioLegend, San Diego, California, USA	rat-IgG-2c, κ-PE	RTK4174/BioLegend, San Diego, California, USA
monocytes, myeloid-derived suppressor cells	rab-anti-S100A9 and sek. goat-anti-rabbit-IgG-Fab-FITC	polyclonal/Institute of Immunology, University of Muenster and Jackson Immuno Research, Ely, Cambridgeshire, UK	rab-IgG and sek. goat-anti-rabbit-IgG-Fab-FITC	Jackson Immuno Research, Ely, Cambridgeshire, UK
T helper cells	CD4-APC	GK1.5/BioLegend, San Diego, California, USA	rat-IgG-2b, κ-APC	A95-1/Biosciences, Franklin Lakes, New Jersey, USA
cytotoxic T cells	CD8a-FITC	53-6.7/BioLegend, San Diego, California, USA	rat-IgG-2a, κ-FITC	R35-95/Biosciences, Franklin Lakes, New Jersey, USA
T cells	CD3-PB	17A2/BioLegend, San Diego, California, USA	rat-IgG-2b, κ-PB	RTK4530/BioLegend, San Diego, California, USA

## Data Availability

The data supporting the findings of this study are available within the article.

## Supplementary Materials

The following are available online at https://www.mdpi.com/2227-9059/9/1/29/s1, Table S1: Therapy scheme and dosages; Figure S2: Exemplary Flow cytometry gating strategy.
