# Supplementary material for: S100A9-Imaging Enables Estimation of Early Therapy-Mediated Changes in the Inflammatory Tumor Microenvironment
Source: Biomedicines. 2021 Jan 3;9(1):29. doi: 10.3390/biomedicines9010029 (PMC7823872; doi:10.3390/biomedicines9010029)
Supplement: Supplementary file 1 [file biomedicines-09-00029-s001.pdf]

Supplementary Table S1. Therapy scheme and dosages. Overview of the different therapy groups with the respective therapy applications.

Therapy	Dosage	Frequency of application	Type of application
Bevacizumab	5 µg/g body weight	twice	i. v. bolus
Doxorubicin	5 µg/g body weight	once	i. v. bolus
0,9 % NaCl	5 µg/g body weight	twice	i. v. bolus

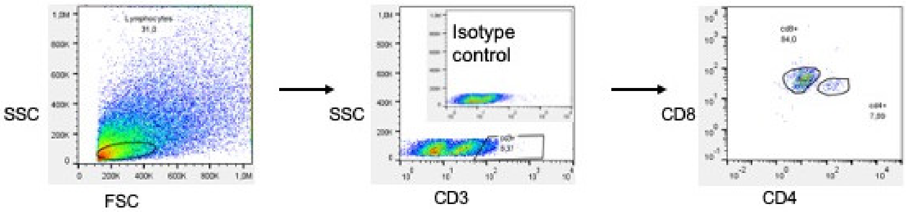

Supplementary Figure S2. Exemplary flow cytometry gating stategy; gating strategy concerning CD4+ and CD8+ cells.
